# Case Report: Premature Ventricular Beats-Induced Chronic Cough and Cough Syncope

**DOI:** 10.3389/fmed.2021.641948

**Published:** 2021-03-10

**Authors:** Yue Hu, Wen Hua, Na Li, Huaqiong Huang

**Affiliations:** Key Laboratory of Respiratory Disease of Zhejiang Province, Department of Respiratory and Critical Care Medicine, Second Affiliated Hospital of Zhejiang University School of Medicine, Hangzhou, China

**Keywords:** chronic cough, syncope, premature ventricular beats, radiofrequency ablation, arrhythmia

## Abstract

**Background:** Chronic cough is a common complaint that in rare cases can be caused by premature ventricular beats (PVCs).

**Materials and Methods:** In this report, we present the case of a healthy 44-year-old female who presented persistent cough and cough syncope that was attributed to PVCs.

**Results:** The cough disappeared after radiofrequency ablation, and no recurrence of arrhythmia or cough was observed.

**Conclusion:** PVCs should be considered a probable cause of chronic cough and cough syncope in differential diagnosis.

## Introduction

Chronic cough, which is defined as a cough lasting for more than 8 weeks, is the most common symptom that accompanies a diverse range of respiratory diseases, non-respiratory conditions, and rarer conditions (1, 2). Various causes of chronic cough have been found, including upper airway cough syndrome, gastroesophageal reflux disease (GERD)/laryngopharyngeal reflux disease, asthma, and non-asthmatic eosinophilic bronchitis (NAEB); however, almost 7% of all chronic coughs are unexplained (3–5). This case report describes a patient with premature ventricular beats (PVCs) presenting with chronic cough and cough syncope.

## Case Presentation

**Figure 1:**
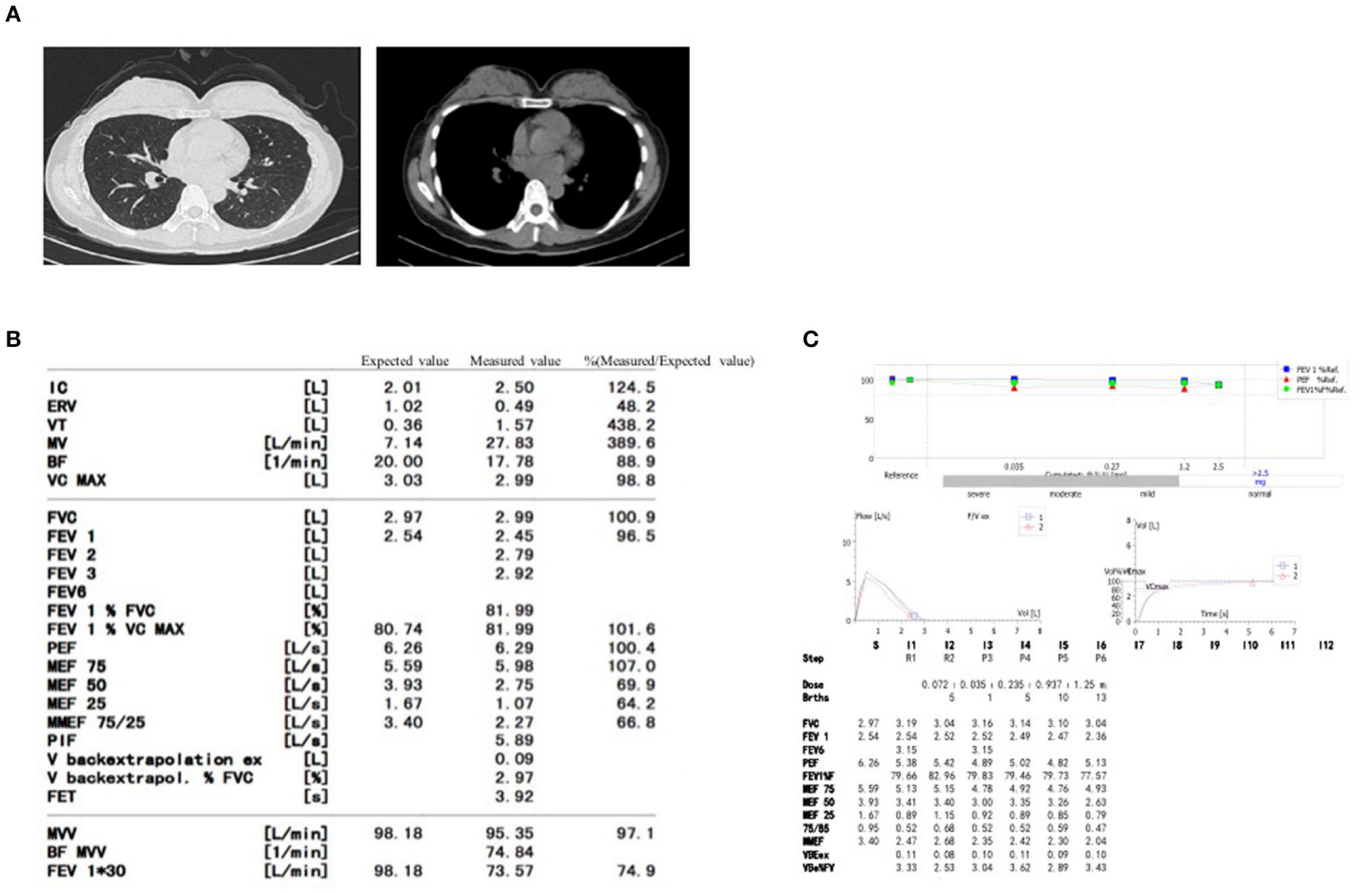
The examination of Chest high-resolution CT **(A)**, pulmonary function **(B)** and methacholine challenge test **(C)** in the patient.

**Figure 2:**
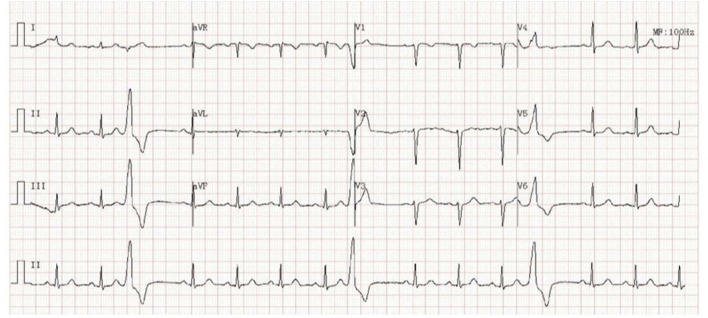
Electrocardiogram (ECG) showing normal sinus rhythm with premature ventricular complexes (PVCs).

**Figure 3:**
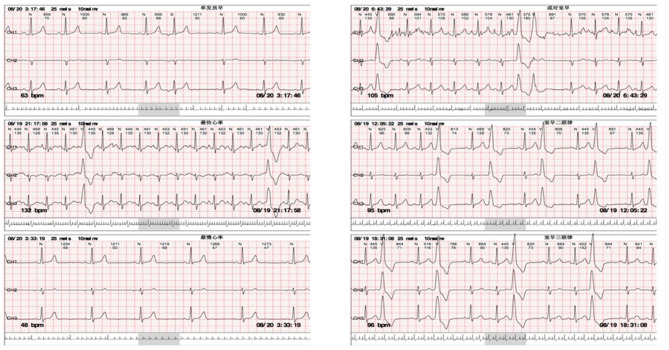
24 h-Holter monitoring showing normal sinus rhythm with premature ventricular complexes (PVCs) during coughing.

**Figure 4:**
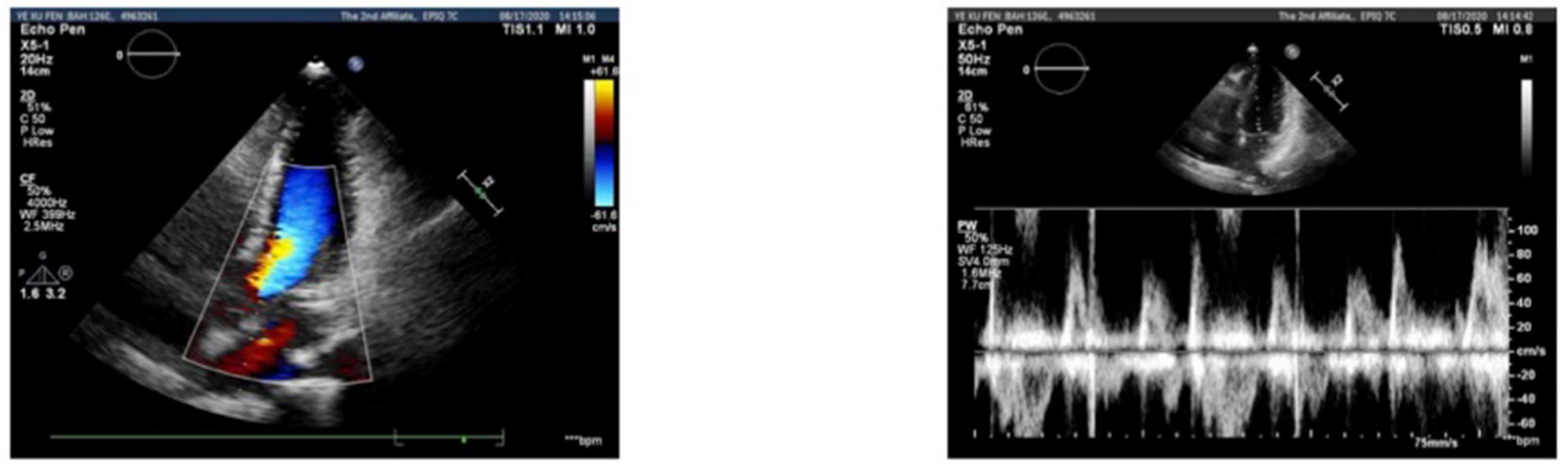
Doppler echocardiogram recordings of blood flow during sinus rhythm and premature ventricular complexes (PVCs).

**Figure 5:**
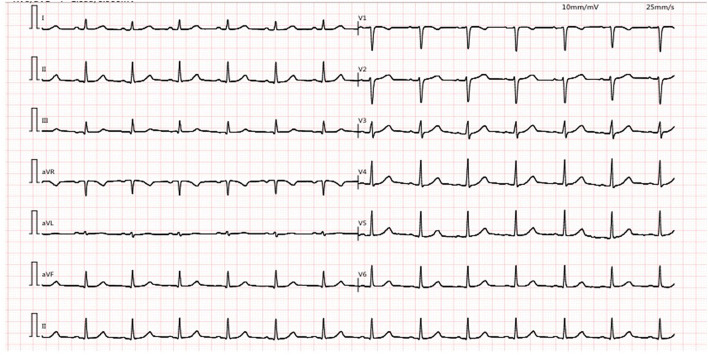
ECG showing sinus rhythm after successful RF application.

This 44-year-old female presented with daily productive cough and expectoration for 4 years and experienced recurrence for more than half a year. The respiratory symptoms were predominantly nocturnal and more severe in the winter. She was diagnosed with chronic cough and was treated with cough medications for cough symptoms, which did not prevent the disease from worsening. Seven months prior, the chest film and spirometry test were almost normal; however, 4 months prior, the cough returned and the patient suffered from cough-related syncope for 3 h. She denied chest pain, chest distress, shortness of breath, heartburn, or acid regurgitation. She had no known allergies and denied smoking. The patient presented no abnormality on physical examination. White blood cell count, eosinophil count, and serum immunoglobulin E (39.1 IU/mL, normal value <100 IU/mL) were normal. Chest high-resolution CT (HRCT) (Figure 1A), pulmonary ventilation and single-breath diffusing capacity of the lung for carbon monoxide were normal. The methacholine challenge test for bronchial hyperreactivity was negative (Figure 1B). In pulmonary function testing, the forced vital capacity (FVC) was 2.99 L (100.9% of predicted value), the forced expiratory volume in 1 s (FEV_1_) was 2.45 L (96.5% of predicted value), and the FEV_1_/FVC ratio was 81.99% (Figure 1C). The patient's electrocardiogram (ECG) showed normal sinus rhythm with frequent premature ventricular beats (PVCs) (Figure 2). The 24 h-Holter ECG monitor showed normal sinus rhythm with 27553 PVCs (Figure 3). The frequent occurrence of PVCs was associated with repetitive coughing. Two-dimensional color echocardiography was normal except for trivial regurgitant flow (Figure 4). Subsequent treatment with oral antiarrhythmic drugs was ineffective. Therefore, radiofrequency ablation of the arrhythmogenic focus was proposed. Surprisingly, cough disappeared after radiofrequency ablation, and no recurrence of arrhythmia or cough was observed (Figure 5).

## Discussion

We report the case of a 44-year-old female who presented to our hospital for PVCs with chronic cough and cough syncope. Symptoms were resolved immediately with the suppression of the PVCs, supporting the causal relationship. The mechanism is uncertain. It has been proposed that PVC-induced cough happens due to the cardiopulmonary reflex (6). Sebastian and his colleagues reported that a patient had premature ventricular complex-induced chronic cough and cough syncope and speculated that the cough reflex was triggered by stimulating sympathetic innervation in the right ventricular outflow tract or pulmonary artery (6). Nimii et al. suggested PVC- induced haemodynamic changes in the pulmonary circulation as factors inducing chronic cough (7). Another mechanism is the cough reflex, which either directly activates C-fibers in the left ventricular wall or distends the pulmonary artery and activates cough receptors as a result of augmented pulmonary blood flow following PVCs (8).

At present, many mechanisms have been reported to be associated with cough syncope (9–12). We suspect that pronounced hypotension and an inappropriate cough-triggered blood pressure-heart rate relationship during PVCs could contribute to syncope and reduce cerebral flow due to vagal modulation (5, 13–15).

Approximately 5% of patients with PVCs present with chronic cough (6). In addition, some less common conditions also occur with chronic cough, such as idiopathic pulmonary fibrosis, eosinophilic bronchitis, and sarcoidosis. These were concluded to be the cause for chronic cough.

## Conclusion

Our patient's case demonstrated PVC-induced cough and cough syncope in a single healthy patient. We suspect that the PVCs might be responsible for coughing in our patient. Even though cough is a rare symptom, it is necessary to explain potential mechanisms between cough and PVC or other arrhythmias. PVCs should be considered a probable cause of chronic cough in the clinical setting.

## Data Availability Statement

The original contributions presented in the study are included in the article/supplementary material, further inquiries can be directed to the corresponding author/s.

## Ethics Statement

The studies involving human participants were reviewed and approved by the Institutional Review Board for Human Studies of Second Affiliated Hospital of Zhejiang University School of Medicine (Hangzhou, China). The patients/participants provided their written informed consent to participate in this study. Written informed consent was obtained from the individual(s) for the publication of any potentially identifiable images or data included in this article.

## Author Contributions

YH examined the patient, ordered the needed tests, interpreted the data, and was a major contributor in finalizing the manuscript. WH interpreted the data and analyzed the results. NL and HH collected the needed data, analyzed and interpreted the results, and was a major contributor in finalizing the manuscript, and submitting it for publication. All authors read and approved the final manuscript.

## Conflict of Interest

The authors declare that the research was conducted in the absence of any commercial or financial relationships that could be construed as a potential conflict of interest.
